# Multidisciplinary treatment for functional neurological symptoms: a prospective study

**DOI:** 10.1007/s00415-014-7495-4

**Published:** 2014-09-20

**Authors:** Benedetta Demartini, Amit Batla, Panayiota Petrochilos, Linda Fisher, Mark J. Edwards, Eileen Joyce

**Affiliations:** 1 Sobell Department, UCL Institute of Neurology, Queen Square, London, WC1N 3BG UK; 2 Department of Psychiatry, San Paolo Hospital and University of Milan, Milan, Italy; 3 Department of Neuropsychiatry, The National Hospital for Neurology and Neurosurgery, UCL Institute of Neurology, Queen Square, Box 19, London, WC1N 3BG UK

**Keywords:** Functional neurological symptoms, Psychogenic neurological symptoms, Conversion disorders, Inpatient programme, Psychotherapy, Physiotherapy

## Abstract

Although functional neurological symptoms are often very disabling there is limited information on outcome after treatment. Here we prospectively assessed the short- and long-term efficacy of an inpatient multidisciplinary programme for patients with FNS. We also sought to determine predictors of good outcome by assessing the responsiveness of different scales administered at admission, discharge and follow-up. Sixty-six consecutive patients were included. Assessments at admission, discharge and at 1 year follow-up (55 %) included: the Health of the Nation Outcome Scale, the Hospital Anxiety and Depression Scale, the Patient Health Questionnaire-15, the Revised Illness Perception Questionnaire, the Common Neurological Symptom Questionnaire, the Fear Questionnaire and the Canadian Occupational Performance Measure. At discharge and at 1 year follow-up patients were also asked to complete five-point self-rated scales of improvement. There were significant improvements in clinician-rated mental health and functional ability. In addition, patients reported that their levels of mood and anxiety had improved and that they were less bothered by somatic symptoms in general and neurological symptoms in particular. Two-thirds of patients rated their general health such as “better” or “much better” at discharge and this improvement was maintained over the following year. Change in HoNOS score was the only measure that successfully predicted patient-rated improvement. Our data suggest that a specialized multidisciplinary inpatient programme for FNS can provide long-lasting benefits in the majority of patients. Good outcome at discharge was exclusively predicted by improvement in the HoNOS which continued to improve over the 1 year following discharge.

## Introduction

Functional neurological symptoms (FNS) represent one of the commonest diagnoses made in outpatient neurology clinics [1]. Long-term follow-up studies report lack of recovery and even worsening of symptoms in one half to two-thirds of patients [2, 3].

To date there are no official guidelines for the treatment of FNS. Different approaches, including pharmacotherapy (mainly antidepressants) [4], psychological therapies (both cognitive-behavioural and psychodynamic) [5, 6], hypnotherapy [7] and physical rehabilitation [8, 9], have been considered helpful in a variable proportion of patients with FNS. Inpatient treatment programmes combining different approaches [10, 11] have also been described. We have recently shown that patients with functional symptoms [11] benefitted from this approach; but the sample was small, and only included patients with functional motor symptoms. In addition, this a retrospective study, based on patients’ own estimation of their disability up to 7 years earlier. In another study, also restricted to functional motor symptoms, McCormack et al. found significant improvement following a multidisciplinary inpatient programme. This was again retrospective and relied on case notes rather than direct patient contact [12]. Recently, Jordbru et al. [13] examined the effect of a 3 week inpatient rehabilitation programme on 60 patients affected by functional gait disorders; they found patients to significantly improve their ability to walk and their quality of life after inpatient rehabilitation compared with the untreated control group. This was the first randomized controlled trial assessing inpatient programme for FNS. Nevertheless, the focus of the programme was physical rehabilitation; occupational therapy and cognitive-behavioural therapy were not provided. In addition, they only included in the study patients with functional gait disorders.

In this study, we examined prospectively the short- and long-term efficacy of an inpatient multidisciplinary programme for patients with functional neurological symptoms of all types. To do this we used a range of clinician- and patient-rated assessment scales to measure mental health, physical symptoms, every-day function and illness perception. We additionally evaluated the responsiveness of these instruments to patients’ own self-report of change to determine whether there were baseline predictors of good outcome.

## Materials and methods

### Subjects

Sixty-six consecutive patients affected by functional neurological symptoms (FNS) treated within a specialized multidisciplinary inpatient programme between January 2010 and May 2012 was included. This was a different patient group to that described in our previous study [11]. All patients were older than 18 years and were able to communicate well in written and spoken English. Ethical approval was obtained from the UCL Institute of Neurology and National Hospital for Neurology Joint Ethics Committee and all patients provided written informed consent to participate.

### Description of the programme

Patients were admitted to the neuropsychiatry inpatient unit of The National Hospital for Neurology and Neurosurgery, Queen Square, London for a 4 week treatment programme. All the patients who accepted programme have an established diagnosis of functional neurological symptoms which has been ascertained by a neurologist and psychiatrist on the basis of clinical presentation and appropriate investigations. To optimize the efficacy of the programme, a few months before the admission patients attend an assessment clinic where they have the opportunity to meet the multidisciplinary team and discuss their diagnosis and the rationale behind the programme. On admission, the treatment plan is individualized and tailored to each patient according to his or her treatment goals. Common features are cognitive-behavioural therapy, occupational therapy, physiotherapy, neuropsychiatry and neurology assessment and input. For further details on the programme please see Saifee et al. [11].

### Assessment

Self-report and clinician-rated assessments were completed by the patients at admission and discharge. At 1 year follow-up, all patients were sent the self-report questionnaires and a psychiatrist conducted a telephone assessment using a semi-structured interview for the clinician-rated scales.

### Clinician-rated assessments

#### Health of the Nation Outcome Scale (HoNOS)

This is a well-validated 12-item instrument for the assessment of psychiatric symptoms, behaviour, impairment and social functioning in patients with mental illnesses. It is the most widely clinician-rated routine outcome measure in British mental health services [14]. Each category is scored on a scale ranging from 0 to 4: 0 = no problem; 1 = minor problem requiring no action; 2 = mild problem but definitely present; 3 = moderately severe problem; 4 = severe to very severe problem. A higher score therefore indicates greater impairment, and the maximum score is 48.

#### Canadian Occupational Performance Measure (COPM)

This is an individualized measure designed for use by occupational therapists to detect change in a patient’s self-perception of occupational performance over time; it is administered via semi-structured interview. This measure is used to help patients identify areas of difficulty in self-care, productivity, and leisure. Following identification of up to five problem areas, patients rate each on a scale from 1 (least important) to 10 (most important). Patients also rate their current level of performance and satisfaction with their performance in each of the five areas on a scale from 1 (with great difficulty or not satisfied) to 10 (with no difficulties or completely satisfied). On re-assessment, patients review their goals and again rate their performance and satisfaction on the goals identified in the initial assessment [15]. The COPM has been shown to be a valid measure of functional outcomes and is sensitive to change. An improvement of two points is regarded as clinically significant [16].

### Self-report assessments

#### Hospital Anxiety and Depression Scale (HADS)

This is a reliable 14-item self-assessment scale developed to detect states of anxiety, depression and emotional distress among patients who were treated for several medical conditions [17]. The items are scored on a 4-point Likert scale, ranging from 0 to 3: score range is 0–42 for the total score.

#### Fear Questionnaire (FQ)

This is a brief 15-item self-report measure of three dimensions of fear (agoraphobia, social phobia, and blood/injury phobia). The FQ is a frequently used measure in anxiety disorder assessment and research and has been used to measure fear in a variety of other clinical populations [18]. The score range is 0–150; higher scores indicate greater agoraphobia, social phobia, and blood/injury phobia.

#### Patient Heath Questionnaire-15 (PHQ-15)

This is a multiple-choice self-report inventory, used as a screening and diagnostic tool for somatic symptoms. It was designed for use in the primary care setting but it is now commonly used in specialized settings [19]. The symptoms inquired include 14 of the 15 most prevalent somatic symptoms listed in the Diagnostic and Statistical Manual of Mental Disorders-fifth edition (DSM-V) criteria for somatization disorder [20]. Each individual symptom is coded as 0 (not bothered at all by this symptom), 1 (slightly bothered by this symptom), or 2 (bothered a lot by this symptom), and the total score ranges from 0 to 30. Higher scores represent worse somatic symptoms.

#### The Common Neurological Symptom Questionnaire (CNSQ)

This is a nine item questionnaire where patients are asked to indicate in the last 4 weeks how bothered have they have been (not at all bothered, slightly bothered, bothered a lot) by a range of common neurological symptoms [21]. Each individual symptom is coded as 0, 1, or 2 as for the PHQ-15, and the total score ranges from 0 to 27 with higher scores meaning more bothered.

#### The Revised Illness Perception Questionnaire (IPQ-R)

This is a reliable and well-validated self-report instrument for assessing cognitive representations of illness. It is a theoretically derived measure comprising sub-scales that provides information about the components that have been found to underlie the cognitive representation of illness. These are: *timeline* *acute/chronic*—the belief about the chronicity of illness, *consequence*—the expected adverse effects and outcomes; *timeline cyclical*—the day to day variability of symptoms; *personal control*—the degree to which symptoms can be self-controlled; *treatment control*—the degree to which symptoms can be helped with treatment; *illness coherence*—understanding about symptoms; *emotional representations*—degree of distress caused by symptoms. Each question is answered on a 1–5 Likert scale, and subscores for each of domain are calculated [22].

Following the methodology of Sharpe et [21], self-assessment of outcome at discharge and 1 year follow-up was established by asking patients to complete a five-point scale (CGI), which asked them to compare their current general health with that before the admission on a five-point scale (“much worse”; “worse”; “not changed”; “better”; “much better”). They were also asked to make the same rating for improvement specifically in their presenting symptoms (IPS).

### Analysis

Statistical analysis was performed using SPSS version 21 (Statistical Package for Social Science). First, we compared the sample of patients on whom we had 1 year follow-up data with the sample of patients on whom these data were missing, using *t* test and *χ* ^2^ test as appropriate, to test whether we had an inclusion bias. We then compared outcomes on the CGI and IPS scales administered at discharge and at 1 year follow-up using the Friedman test. The CGI score was used to define two groups: good outcome (CGI: “much better” or “better”) and poor outcome (CGI: “not changed”, “worse”, “much worse”). Differences in the mean scores of each scale were assessed using repeated measures ANOVA over the following time points: on admission, on discharge and at 1 year follow-up. Correlation analyses were undertaken using Pearson or Spearman’s correlation coefficient as appropriate.

## Results

Sixty-six patients were assessed at admission and discharge. The median age was 43.7 years (SD 14.7) and 70.2 % were females. The dominant symptoms on admission were movement disorders (50.5; 16.1 % dystonia, 8.9 % jerks, 8 % tremor, gait disturbances 7.5 % and 10 % mixed), psychogenic non-epileptic seizures (21.2 %), weakness (18.8 %) and sensory symptoms (9.5 %). The mean illness duration was 4.8 years (DS 3.2 years, range 1–8 years).

At the time of admission, 33 % of patients had a comorbid mood disorder (mainly depression), 39 % an anxiety disorder; 16 % had a diagnosis of personality disorders. None of the patients had a diagnosis of psychosis.

At the time of admission, 71 % of patients had left their job because of illness, and 95 % were in receipt of health-related financial benefits.

### Symptoms

Table 1 shows that for the group as a whole there were significant improvements in: HoNOS, HADS, PHQ15, and CNSQ. The effect sizes were large for HoNOS, medium for PHQ and CNSQ and small for HADS.

**Table 1:** Group mean (standard deviation) changes in assessment scores between admission and discharge

	*N*	Admission	Discharge	*t* statistic	Effect size (Cohen’s *d*)
HoNOS^a^	64	9.7 (4.1)	6.4 (3.9)	10.0 *p* < 0.001	0.84
HADS^b^	62	15.8 (8.5)	13.3 (8.2)	3.0 *p* = 0.004	0.26
FEAR^c^	66	35.1 (26.9)	31.3 (26.8)	1.8 *p* = 0.07	0.14
PHQ15^d^	64	12.6 (5.5)	9.8 (5.1)	5.4 *p* < 0.001	0.53
CNSQ^e^	64	8.0 (4.3)	6.0 (3.8)	4.1 *p* < 0.001	0.50
IPQ-R timeline acute/chronic^f^	61	18.4 (4.4)	16.2 (4.4)	4.5 *p* < 0.001	0.51
IPQ-R timeline cyclical^f^	61	13.4 (3.9)	13.3 (4.2)	0.1 *p* = 0.90	0.02
IPQ-R consequences^f^	61	22.7 (4.9)	21.5 (4.4)	2.4 *p* = 0.02	0.26
IPQ-R personal control^f^	61	21.5 (3.6)	22.5 (4.1)	1.8 *p* = 0.07	0.27
IPQ-R treatment control^f^	61	18.4 (2.9)	17.7 (4.6)	1.1 *p* = 0.25	0.18
IPQ-R illness coherence^f^	61	13.5 (5.0)	17.5 (4.6)	5.6 *p* < 0.001	0.84
IPQ-R emotional representation^f^	61	20.5 (5.6)	18.7 (5.5)	2.7 *p* = 0.009	0.32

*HoNOS* Health of the Nation Outcome Scale; *HADS* Hospital Anxiety and Depression Scale; *FEAR* Fear Questionnaire; *PHQ-15* Patient Heath Questionnaire-15; *CNSQ* Common Neurological Symptom Questionnaire; *IPQ-R* Revised Illness Perception Questionnaire

^a^Score range = 0–48. Higher score indicates greater impairment

^b^Score range = 0–42. Higher score indicates grater anxiety and depression

^c^Score range = 0–150. Higher scores indicate greater agoraphobia, social phobia, and blood/injury phobia

^d^Score range = 0–30. Higher scores represent worse somatic symptoms

^e^Score range = 0–27. Higher scores indicate more bothered by common neurological symptoms

^f^Score range = 0–30. Higher scores indicate stronger perception of illness chronicity, a cyclical timeframe and negative consequences, and greater emotional distress; lower scores indicate low perceived personal and treatment control over the illness and less understanding of the illness. The IPQ-R scoring information are available online (www.uib.no/ipq)

**Fig. 1:**
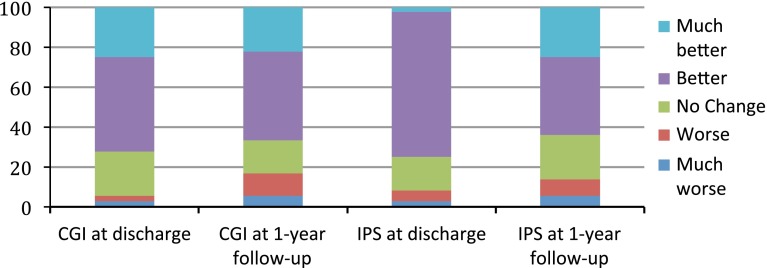
Patient-rated outcome at discharge and at 1 year follow-up on CGI and IPS Likert scales. *CGI* clinical global improvement; *IPS* improvement in presenting symptom. At discharge 2.8 % patients rated their general health such as “much worse”, 2.8 % such as “worse”, 22.2 % such as “no change”, 47.2 % such as “better”, 25 % such as “much better” on the CGI and 2.8 % rated their main symptoms such as “much worse”, 5,5 % such as “worse”, 16.7 % such as “no change”, 72.8 % such as “better” and 2.2 % such as “much better” on the IPS. At 1 year follow-up 5.6 % patients rated their general health such as “much worse”, 11.1 % such as “worse”, 16.7 % such as “no change”, 44.4 % such as “better”, 22.2 % such as “much better” on the CGI and 5.6 % rated their main symptoms such as “much worse”, 8.3 % such as “worse”, 22.2 % such as “no change”, 38.9 % such as “better” and 25 % such as “much better” on the IPS

At discharge 66.2 % patients rated their general health such as “better” or “much better” on the CGI and 75.0 % rated their main symptoms as “better” or “much better” on the IPS (Fig. 1). A binary logistic regression analysis was conducted to examine whether the variables that improved with treatment predicted, at baseline, the self-rated outcome on the CGI. The combination of variables classified correctly 70.5 % of patients. The overall model was not significant (*χ* ^2^ = 2.18, *df* = 4, *p* = 0.70) and only 4.9 % of the variance in outcome could be explained (Nagelkerke R2). This was repeated for change scores of the same variables. The classification was improved (75 %), the model fit was significant (*χ* ^2^ = 9.62, *df* = 4, *p* = 0.047) and 20.8 % of the variance in outcome was explained. Of the four variables entered, only change in HoNOS score was a significant predictor of self-rated outcome (Wald (1) = 4.52, *p* = 0.033).

### Function

The Canadian occupational performance measure (COPM), a measure of self-perceived change, was completed on 51 patients. There was a mean improvement of 3.12 (1.78) points for performance and 3.90 (2.06) points for satisfaction between baseline and discharge for the whole group. Change in neither performance nor satisfaction subscales predicted self-rated improvement on CGI (model fit: *χ* ^2^ = 5.30, *df* = 2, *p* = 0.07; performance: Wald (1) = 1.50, NS; satisfaction: Wald (1) = 0.02, NS).

### Illness perception

For the whole group, there were significant changes between admission and discharge on the IPQR timeline acute/chronic; illness coherence; emotional representations and consequences subscales. Binary logistic regression analyses for the admission scores on CGI outcome showed that 69 % of the patients were classified correctly, the overall model was not significant (*χ* ^2^ = 1.81, *df* = 4, *p* = 0.77) and only 3.8 % of the variance could be explained (Nagelkerke R2). Similarly, when change scores were entered, 67 % patients were correctly classified, the overall model was not significant (*χ* ^2^ = 6.52, *df* = 4, *p* = 0.16) and 14 % of variance was explained.

### Long-term outcome

Thirty-six (55 %) patients returned their questionnaires and were interviewed by telephone approximately, 12 months after discharge (range: 10-15 months). At discharge, 72.2 % rated their general health as “better” or “much better” on the CGI and 80.5 % rated their main symptoms as “better” or “much better” on the IPS. At 12 months, these figures were 66.6 and 63.9 %. We compared how the CGI and IPS self-rating had changed from discharge to follow-up; 13.9 and 25 %, respectively rated themselves better at discharge but unchanged at follow-up; 8.3 and 8.3 %, respectively rated themselves as unchanged at discharge and better at follow-up.

Compared to those who did not complete the 12 month assessment, the patients who were followed up at 12 months had lower scores on the HoNOS (*t*(62) = 2.22, *p* = 0.030 and the FEAR questionnaire (*t*(64) = 3.32, *p* = 0.001) at admission; they also rated their symptom fluctuations as having a less deleterious impact (IPQ timeline cyclical *t*(63) = 3.33; *p* = 0.001).

Table 2 shows repeated measures analyses of variance over the three time points (admission, discharge and 12 months follow-up). A significant time course effect was found for: HoNOS, PHQ15, CNSQ and several subscales of the IPQ (timeline acute/chronic, illness coherence, emotional representation). There was a strong trend for a significant time course effect for HADS. For these variables, pairwise contrasts between admission and discharge scores showed significant improvements in all of these variables. Pairwise contrasts between discharge and 12 months were significant for improvement in HoNOS (*t*(30) = 3.24; *p* = 0.003; *d* = 0.69) and deterioration in timeline acute/chronic IPQR subscale (*t*(33) = 2.83, *p* = 0.008; *d* = 0.61).

**Table 2:** Repeated measures analyses of variance over the three time points (admission, discharge and 12 months follow-up)

	Admission	Discharge	1 year	*F* statistic	Effec*t* size (partial eta squared)	Comparisons: (a) admission *v* discharge (b) discharge *v* 1 year
HoNOS	8.7	5.6	4.0	*F*(2,30) = 32.42 *p* < 0.001	0.52	(a) *t*(35) = 7.6; *p* < 0.001 (b) *t*(30) = 3.2; *p* = 0.003
HADS	14.3	11.3	12.6	*F*(2,35) = 2.75 *p* = 0.07	0.07	(a) *t*(35) = 2.7; *p* = 0.011 (b) NS
FEAR	25.8 (15.5)	24.9 (19.0)	27.4 (20.9)	*F*(2,35) = 0.43 *p* = 0.65	0.01	
PHQ15	11.7	9.2	9.2	*F*(2,35) = 5.21 *p* = 0.001	0.13	(a) *t*(35) = 3.9; *p* < 0.001 (b)NS
CNSQ	7.5	5.4	5.7	*F*(2,35) = 5.20 *p* = 0.008	0.23	(a) *t*(35) = 2.9; *p* = 0.006 (b) NS
IPQ-R timeline Acute/chronic	18.8	16.7	19.4	*F*(2,32) = 4.66 *p* = 0.01	0.13	(a) *t*(34) = 3.0; *p* = 0.005 (b) *t*(30) = -2.8; *p* = 0.008
IPQ-R timeline cyclical	11.9	12.9	12.8	*F*(29) = 0.63 *p* = 0.54	0.02	
IPQ-R consequences	23.2	22.2	21.6	*F*(2,29) = 1.35 *p* = 0.27	0.04	
IPQ-R personal control	21.5	23.4	21.5	*F*(2,29) = 1.89 *p* = 0.16	0.06	
IPQ-R treatment control	18.4	18.0	17.8	*F*(2,29) = 0.32 *p* = 0.73	0.01	
IPQ-R illness coherence	13.4	17.88	17.0	*F*(2,29) = 13.32 *p* < 0.001	0.32	(a) t(31) = 4.4 *p* < 0.001 (b) NS
IPQ-R emotional representation	20.6	18.79	17.8	*F*(2,29) = 3.65 *p* = 0.03	0.11	(a) t(31) = 2.1; *p* = 0.044 (b) NS

*HoNOS* Health of the Nation Outcome Scale, *HADS* Hospital Anxiety and Depression Scale, *FEAR* Fear Questionnaire, *PHQ-15* Patient Heath Questionnaire-15, *CNSQ* Common Neurological Symptom Questionnaire, *IPQ-R* Revised Illness Perception Questionnaire

The COPM was completed on 27 patients at 12 months follow-up. These patients showed an improvement from admission of 3.30 (2.59) for performance and 4.12 (2.72) for satisfaction. When changes between admission and discharge were compared with discharge and 12 months there were no differences (performance: *t*(34) = 0.58; satisfaction: *t*(34) = 0.58). Illness perceptions for the group that was followed up showed a change from admission for illness coherence (*t*(32) = −4.64; *p* ≤ 0.001) and emotional representation (*t*(32) = 2.24; *p* = 0.032).

## Discussion

We studied a large group of patients with functional neurological symptoms who were consecutive admissions for a 4 week multidisciplinary treatment programme. We found significant improvements in clinician-rated mental health and functional ability. In addition, patients reported that their levels of mood and anxiety had improved and that they were less bothered by somatic symptoms in general and neurological symptoms in particular.

The effect size for these improvements was greatest for the HoNOS. The HoNOS is a clinician-rated scale devised to assess outcome in people with mental illness and incorporates brief assessments of mental health, social function and behaviour [14]. It is used routinely in the UK, New Zealand and Australia and increasingly so in other European countries and Canada. A comprehensive review [23] concluded that the psychometric properties of the HoNOS are ‘adequate or better’ indicating that it can be used for monitoring outcomes following treatment. Patients with functional symptoms are prone to many aspects of disability captured by the HoNOS, e.g. mood, cognitive and functional impairments. Our study suggests that the HoNOS is a suitable instrument for detecting meaningful improvement following treatment for functional neurological symptoms.

The HoNOS, being a clinician rated scale, does not take into account the patient perspective. We therefore, asked patients to rate their outcome using the CGI which asks them to state whether they are “much worse”; “worse”; “not changed”; “better“; “much better”. We then classified patients as improved if they rated themselves as “much better” or “better. Sixty-six percent of patients were classified as improved using this scheme. The change on HoNOS rating following treatment was the only assessment scale able to predict with reasonable accuracy those who rated themselves as improved and not improved at discharge from hospital. This suggests that the HoNOS tallies well with the patients’ own opinion of their response to treatment, a further validation of the use of the scale for assessing outcomes.

Self-reported improvements in mood and anxiety measured by the HADS and the degree of distress over symptoms measured by PHQ and CSNQ did not predict whether the patients felt they were better or not at the end of treatment. There were significant improvements in these measures across the group but the effect sizes were small to medium suggesting that these measures were not sensitive enough to self-perceived change. The COPM scores also failed to predict whether patients felt they were better or not. This is a clinician-guided scale enabling patient to set goals and rate their change in performance and their satisfaction. As a change in 2 points is regarded as clinically meaningful, our patient group clearly benefitted from the treatment as they reported a mean of 3 and 4-point change in performance and satisfaction, respectively. Similarly, there were significant changes in the perception of their symptoms following treatment; overall patients understood their symptoms better and felt they were less permanent and disabling. Again these changes did not predict whether they felt better or not.

Just over 50 % of patients were reassessed 12 months after discharge. Despite these patients having lower HoNOS scores at admission, compared to the group that were not followed-up, their pattern of improvement at discharge from the treatment programme was virtually identical to the group as a whole. These improvements remained stable over the 12 months following discharge for all self-rated measures, and their HoNOS scores continued to improve.

These data are consistent with the results of retrospective studies of functional movement disorders. For example, Saifee et al. in a different smaller population of patients who completed our programme between 2006 and 2008 [11], and Williams et al. [24], assessing a different hospital based programme, found that functional motor symptoms improved in the majority of patients. Our study extends these results by employing a more accurate prospective design, examining long-term outcome by directly contacting the patients 12 months after discharge, and including patients with a range of functional neurological symptoms.

Previous studies have shown that treatments used for FNS, such as cognitive-behavioural therapy [5, 6] or medications [4], often provide an initial improvement in symptoms but benefits are not maintained in the long-term. In fact, it has been widely described that patients with FNS have a high frequency of relapse or chronicity of the symptoms [25, 26]. Our study suggests that a multidisciplinary approach is important for sustained long-term improvement.

A significant problem for the management of functional neurological symptoms in the UK is that local services are usually unable to accept patients for initial or post-discharge continuing treatment because of the level of demand for their service from neurological patients with organic disorders. An MDT service such as ours is labour-intensive and therefore expensive. Ideally, to optimize the effectiveness of an MDT provision, patients who are likely to benefit from MDT therapy should be identified as early as possible in the care pathway. This is underlined by the finding by Jordbru et al. [13] that physiotherapy-based rehabilitation alone may be effective for some. Unfortunately, none of measures we used at baseline, including the HoNOS, predicted response to treatment as perceived by patients. Our current data therefore suggests that none of these measures could be used to identify which patients are likely to benefit. Alternatively, our measure of patient satisfaction may have been too crude and a dimensional scale such as the CORE-OM may be a better self-assessment tool. This has been validated for patients undergoing therapy and has the advantage of capturing several domains at once including well-being, symptoms and functioning.

### Limitations

We acknowledge the limitations of our study: first, we only achieved 1 year follow-up data on 55 % of patients. Second, there are other potential predictors of good or poor outcome that we did not measure [2]; these might be social such as the receipt of health-related financial benefit and the work situation, or psychological such as the presence of dissociative symptoms and the presence of alexithymia (two aetiological markers of FNS) [27, 28]. Third, the absence of a control group makes it difficult to know to what extent the improvement observed in our patients represented a specific response to treatment intervention.

## Conclusions

Our data suggest that a specialized multidisciplinary inpatient programme for FNS can provide long-lasting benefits in the majority of patients. Good outcome at discharge, as rated by the patient, was only predicted by improvement in the HoNOS which continued to improve over the 1 year following discharge. The HoNOS appears to be a suitable tool for the detection of meaningful improvement following multidisciplinary treatment.
